# Opercular Perivascular Space Mimicking a Space-Occupying Brain Lesion: A Short Case Series

**DOI:** 10.3390/diagnostics15121486

**Published:** 2025-06-11

**Authors:** Roberts Tumelkans, Cenk Eraslan, Arturs Balodis

**Affiliations:** 1 Faculty of Medicine, Riga Stradins University, LV-1007 Riga, Latvia; roberts.tumelkans@gmail.com; 2 Radiology Department, Ege University, 35040 Bornova, Turkey; eraslancenk@gmail.com; 3 Institute of Diagnostic Radiology, Pauls Stradins Clinical University Hospital, LV-1002 Riga, Latvia; 4 Department of Radiology, Riga Stradins University, LV-1007 Riga, Latvia

**Keywords:** opercular perivascular space, magnetic resonance imaging, edema

## Abstract

A newly recognized fourth type of perivascular space has recently been described in the radiological literature. Despite its growing relevance, many radiologists are still unfamiliar with its imaging characteristics, often leading to misinterpretation as cystic neoplasms. Due to its potential for diagnostic confusion, further studies are necessary—particularly those incorporating high-quality imaging examples across various presentations—to facilitate accurate recognition and classification. Perivascular spaces (PVSs) of the brain are cystic, fluid-filled structures formed by the pia mater and located alongside cerebral blood vessels, particularly penetrating arterioles, venules, and capillaries. Under normal conditions, these spaces are small (typically <2 mm in diameter), but in rare instances, they may become markedly enlarged (>15 mm), exerting a mass effect on adjacent brain tissue. This newly identified fourth type of PVS is found in association with the M2 and M3 segments of the middle cerebral artery, typically within the anterior temporal lobe white matter. It may mimic low-grade cystic tumors on imaging due to its size and frequent presence of surrounding perifocal edema. We present two adult male patients with this rare PVS variant. The first patient, a 63-year-old, had a brain magnetic resonance imaging scan (MRI) that revealed a cystic lesion in the white matter of the right temporal lobe anterior pole, near the middle cerebral artery M2 segment, with perifocal vasogenic edema. The second patient, a 67-year-old, had a brain MRI that showed a cystic lesion in the white matter and subcortical region of the right temporal lobe anterior pole, with minimal surrounding gliosis or minimal edema. The cystic lesions in both patients remained unchanged over time on follow-up MRI. These cases illustrate the radiological complexity of this under-recognized entity and emphasize the importance of differential diagnosis to avoid unnecessary intervention.

**Figure 1:**
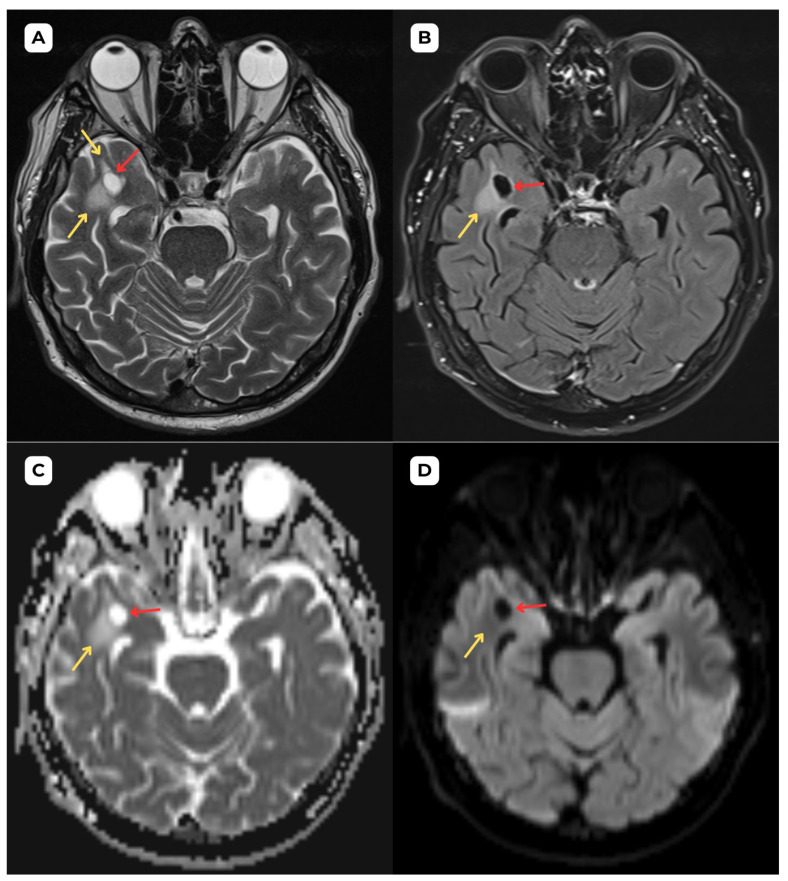
**First patient:** Brain MRI of a 63-year-old male. (**A**) **Axial T2-weighted sequence.** A cystic lesion (**red arrow**) is seen in the anterior part of the superior temporal gyrus of the right temporal lobe, measuring 1.1 × 0.8 cm, accompanied by perifocal edema (**yellow arrows**) in the surrounding white matter. (**B**) **Axial T2-FLAIR after contrast** image at the corresponding level demonstrates improved visualization of the cerebrospinal fluid (CSF) content of the lesion (**red arrow**) and highlights the perifocal edema (**yellow arrow**) more clearly, without contrast enhancement. (**C**) The **ADC map shows a high value with no diffusion restriction on** (**D**) **DWI-B1000, where changes show no signs of potential malignancy, most likely benign structural changes.** The most likely differential diagnosis is opercular (type IV) perivascular space. **Red arrow—opercular (type IV) perivascular space; yellow arrow—surrounding edema.** Perivascular spaces of this type (IV) were first clearly documented with MRI imaging in 2020 [1,2], but there is still insufficient documentation of radiological characteristics with vasogenic edema. Type IV PVSs are typically found in the anterior temporal lobe and are closely associated with the middle cerebral artery M2 and M3 segment branches in the white matter of the brain. These PVSs are frequently associated with perifocal edema, likely due to the region’s loose white matter structure and increased interstitial fluid permeability around small penetrating vessels. It should be emphasized that the presence of perifocal edema does not necessarily indicate an active pathological process; it may represent a reactive or structural phenomenon without clinical significance, particularly in elderly individuals [3,4]. Perifocal edema surrounding an opercular perivascular space can range from minimal to very profound and is found in roughly 80% of cases of type IV PVS [5]. In this case, the first patient had more pronounced perifocal edema in both the initial MRI examination (Figure 1 and Figure 2) and the one-year follow-up MRI (Figure 3) than the second patient (Figure 4, Figure 5 and Figure 6).

**Figure 2:**
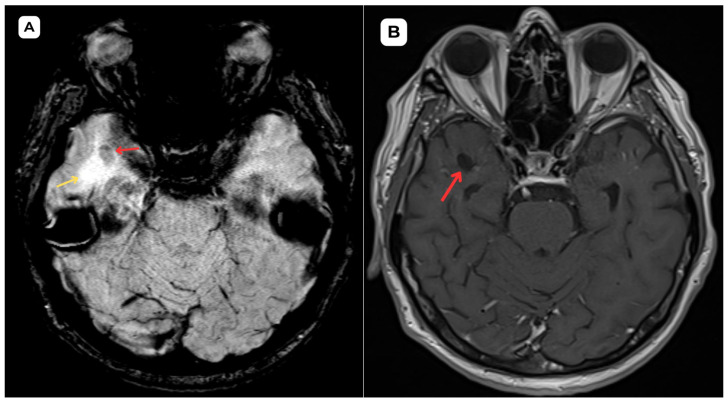
**First patient:** Brain MRI of a 63-year-old male. (**A**) **SWI—susceptibility-weighted imaging, axial.** No evidence of blood products or hemosiderin is seen, providing evidence of the non-hemorrhagic, benign nature of the cystic lesion. (**B**) **The T1 axial post-contrast sequence** shows no abnormal contrast enhancement in the cystic lesion, with no features suggestive of malignancy or disruption of the blood–brain barrier. **Red arrow**—opercular (type IV) perivascular space; yellow arrow—surrounding edema. In magnetic resonance imaging, the signal intensity of the opercular perivascular spaces themselves is identical to that of cerebrospinal fluid in all sequences—hypointense on T1W1 and FLAIR, hyperintense on T2W1, and the ADC value corresponds to cerebrospinal fluid [6,7]. In the images, these PVSs are located very close to the branches of the middle cerebral artery, which come into contact with the cerebral cortex, with MRI showing regional cortical thinning in the brain [6,7]. The presence of cerebrospinal fluid intensity tracts in the images is also useful as a radiological criterion for the diagnosis of perivascular spaces [1]. All of the above-mentioned features help distinguish opercular perivascular spaces from neuroglial tumors with characteristic perifocal edema.

**Figure 3:**
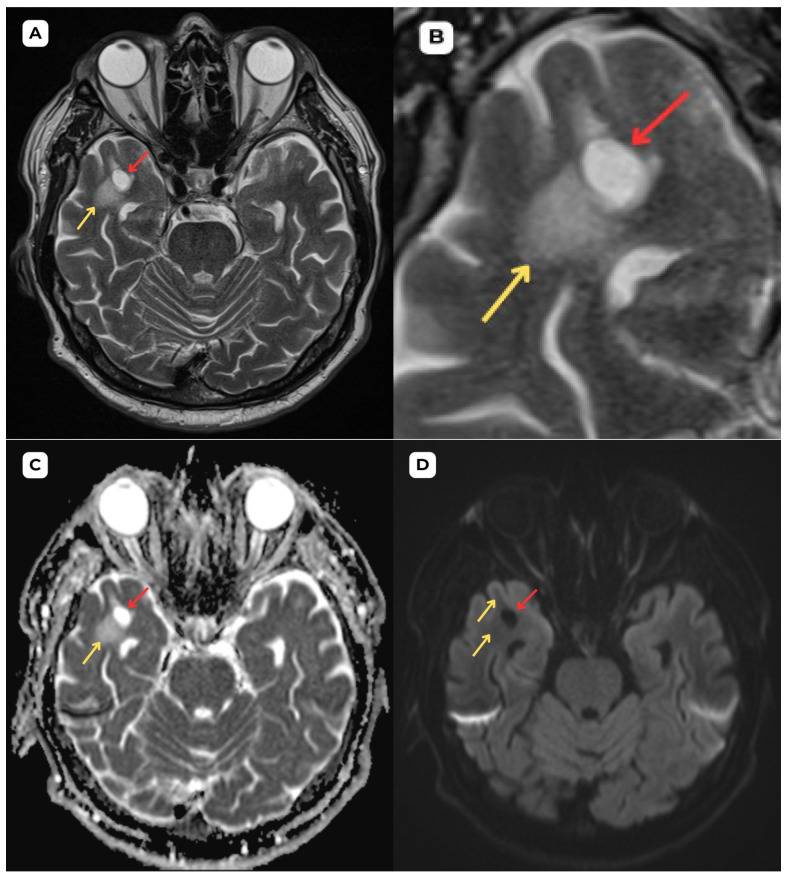
**First patient:** One-year follow-up brain MRI of a 63-year-old male. (**A**) **Axial T2-weighted sequence and** (**B**) **axial T2-weighted sequence zoomed in.** The one-year follow-up shows no changes in the cystic lesion or surrounding structures, supporting the diagnosis of a benign process, consistent with an opercular perivascular space (type IV). The surrounding edema seen previously has remained stable, with no increase in size or signal intensity, further suggesting the lesion’s non-progressive, benign nature. **One-year follow-up shows no changes in** (**C**) **high ADC map values and** (**D**) **no diffusion restriction on DWI B1000. Red arrow—**opercular (type IV) perivascular space; yellow arrow—surrounding edema.

**Figure 4:**
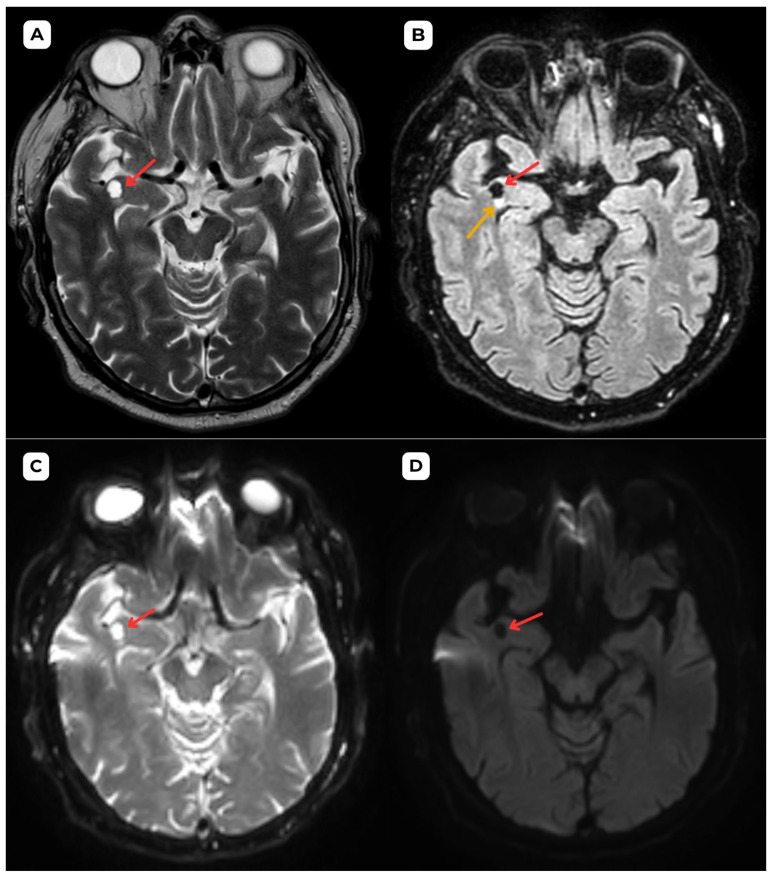
**Accompanying second patient**: Brain MRI of a 67-year-old male. (**A**) **Axial T2-weighted sequence.** A cystic lesion (**red arrow**) located in the upper–middle frontal gyrus, anterior part of the right temporal lobe, measuring 0.8 × 0.7 cm and up to 1.25 cm AP, accompanied by very minimal edema or gliosis, in contrast with the MRI findings in the first patient case. (**B**) **The axial T2-FLAIR sequence** shows the lesion in the same location with better visualization of the CSF contents and emphasizes the very minimal hyperintense perifocal gliosis or edema (yellow arrow). (**C**) **The ADC map displays a high value and no diffusion restriction on** (**D**) **DWI.** Similarly to the first patient, these changes show no signs of potential malignancy. **Red arrow—**opercular (type IV) perivascular space; yellow arrow—surrounding edema. In both of these cases, MRI images were acquired with the Siemens “MAGNETOM Sola” 1.5T system. In both cases, the cerebrospinal fluid signal is visible in all MRI sequences. In MRI imaging, opercular perivascular spaces can also be differentiated based on their appearance: round, oval, or tubular, well-defined cystic structures that do not contain calcifications, hemorrhagic elements, or high-protein content structures. In both cases, opercular perivascular spaces appear round (Figure 1, Figure 2, Figure 3, Figure 4, Figure 5 and Figure 6). These formations do not enhance with contrast, which also helps to distinguish them from other pathologies. In both of the cases, no contrast enhancement was seen (Figure 1, Figure 2, Figure 3, Figure 4, Figure 5 and Figure 6). It is important to highlight the pathognomonic feature—visualization of the perforating central artery of the perivascular space—using TOF combined with 3D CISS sequences [8].

**Figure 5:**
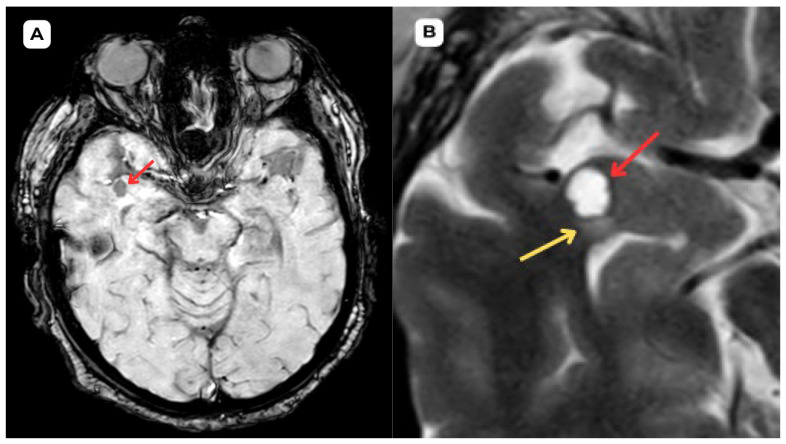
**Accompanying second patient**: Brain MRI of a 67-year-old male. (**A**) **Susceptibility-weighted imaging (SWI), axial.** A cystic lesion (**red arrow**) is seen without any hemosiderin or blood products surrounding it. (**B**) **The axial T2-weighted sequence, zoomed in,** demonstrates better visualization of the very minimal perifocal edema/gliosis (yellow arrow) around the cystic lesion (red arrow) in the white matter. **Red arrow—opercular (type IV) perivascular space; yellow arrow—surrounding edema.** Since low-grade neuroglial tumors show similar signals in standard MRI sequences, functional MRI, MR spectroscopy, and dynamic perfusion—which are based on tumor-induced metabolic changes and molecular mechanisms—play an important role in their diagnosis [9]. Researchers propose using fluid-suppressed Amide Proton Transfer-weighted imaging to aid in the differential diagnosis of vast T2/FLAIR hyperintensity zones in anterior temporal perivascular spaces, which are primarily caused by glial tumors [10]. The method is based on detecting amide signal intensity—in tumor tissues, an increase in APTw signal is observed, whereas in the case of enlarged perivascular spaces, the images do not demonstrate an increase in signal [10]. A publication describes three clinical cases in which the use of this method influenced an early change in the diagnosis from a primary neoplastic disease to a perivascular space [10].

**Figure 6:**
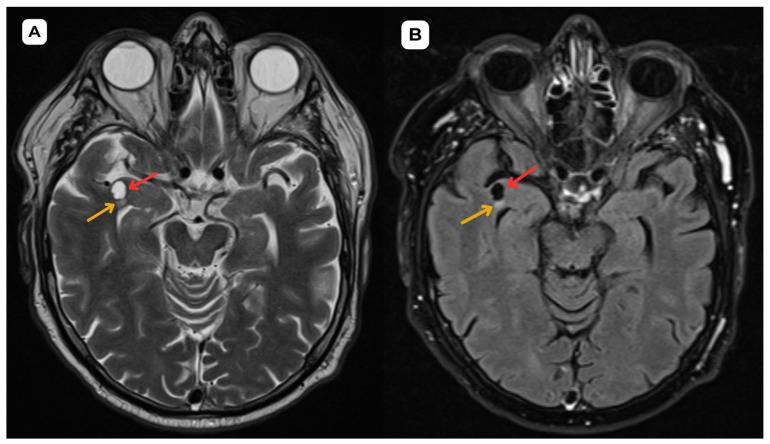
**Accompanying second patient:** One-year follow-up brain MRI of a 67-year-old male. (**A**) **The T2-weighted sequence and** (**B**) **T2-FLAIR one-year follow-up MRI demonstrate no changes in the cystic lesion (red arrows) and surrounding structures.** The minimal perifocal edema or gliosis (yellow arrows) has remained stable; no changes in size or signal intensity can be seen. These findings serve as an important criterion in uncertain cases of PVS; they are likely of a benign nature and consistent with an opercular (type IV) perivascular space. **Red arrow—opercular (type IV) perivascular space; yellow arrow—surrounding edema.** The stability of the lesion and surrounding tissue over the one-year period is reassuring. However, the need for continued monitoring in such cases remains a subject of discussion, as some studies suggest that these lesions tend to remain stable and do not require further follow-up unless there are significant changes [1]. For example, in a study by McArdle et al., 18 patients with opercular perivascular cysts were analyzed. Of the 13 patients who underwent follow-up over a period ranging from 2 months to 10 years, 11 showed no change in cyst size, and only one patient demonstrated a slight increase over a 7-month period [1]. These findings support the notion of long-term structural stability. Additionally, sources such as Radiopaedia.org highlight that opercular (type IV) perivascular spaces, when presenting with typical features, are considered benign and often do not require further imaging unless clinical symptoms or atypical imaging features are present. On the other hand, several authors suggest that such lesions, once identified correctly, typically require only follow-up imaging for monitoring [11,12]. Contrary to the generally stable nature of enlarged perivascular spaces over time, serial MRI studies show a prolonged increase in the size of low-grade neuroglial tumors, averaging 4.1 mm per year [13]. In this case, the aforementioned changes have remained stable over a one-year follow-up MRI. Literature on perivascular spaces, particularly type IV opercular perivascular spaces, is limited in documenting cases with or without surrounding vasogenic edema, and there are not many publications with detailed and structured radiologic images, especially zoomed-in images. This highlights the need for further publications that can provide a clear radiological depiction of such cases. Further studies and imaging examples are necessary to improve diagnostic accuracy and provide a comprehensive understanding of these benign structures.

## Data Availability

The original contributions presented in this study are included in this article; further inquiries can be directed to the corresponding author.
